# Healthier food choices as a result of the revised healthy diet programme Krachtvoer for students of prevocational schools

**DOI:** 10.1186/1479-5868-9-60

**Published:** 2012-05-24

**Authors:** Kathelijne MHH Bessems, Patricia van Assema, Marloes K Martens, Theo GWM Paulussen, Lieke GM Raaijmakers, Mark de Rooij, Nanne K de Vries

**Affiliations:** 1 NUTRIM School for Nutrition, Toxicology, and Metabolism, Department of Health Promotion, Maastricht University Medical Centre+, P.O. Box 616, 6200, MD, Maastricht, The Netherlands; 2 ResCon, Rijswijkstraat 175, 1062, EV, Amsterdam, the Netherlands; 3 TNO (Netherlands Organisation of Applied Scientific Research) Quality of Life, Leiden, the Netherlands, Postbus 2215, 2301, CE, Leiden, The Netherlands; 4 Psychological Institute, Methodology and Statistics group, Leiden University, Wassenaarseweg 52, 2333, AK, Leiden, the Netherlands; 5 CAPHRI School for Public Health and Primary Care, Department of Health Promotion, Maastricht University Medical Centre+, P.O. Box 616, 6200, MD, Maastricht, The Netherlands

**Keywords:** School programme, Nutrition, Dietary effects

## Abstract

**Background:**

Krachtvoer is a Dutch healthy diet programme for prevocational schools, developed in 2001 and revised for a broader target group in 2007, based on the findings of an evaluation of the first version. The goal of this study was to report on the short- and longer-term total and subgroup effects of the revised programme on students’ fruit, fruit juice, breakfast, and snack consumption.

**Methods:**

Schools were randomized to the experimental condition, teaching the Krachtvoer programme, or to the control condition teaching the regular nutrition lessons. Self-reported consumption of fruit, fruit juice, breakfast and snacks was measured at baseline directly before programme implementation, one to four weeks after finishing programme implementation, and after six months. Mixed linear and logistic regression analyses were conducted.

**Results:**

In total 1117 students of 13 experimental schools and 758 students of 11 control schools participated in the study. Short- and longer-term favourable intervention effects were found on fruit consumption (mean difference between experimental and control group 0.15 servings at both posttests). Regarding fruit juice consumption, only short-term favourable effects were revealed (mean difference between experimental and control group 0.05 glasses). Intervention effects on breakfast intakes were limited. No changes in snack frequency were reported, but students made healthier snack choices as a result of the programme. Some favourable as well as unfavourable effects occurred in subgroups of students.

**Conclusions:**

The effects on fruit consumption and snack choices justify the current nationwide dissemination of the programme. Achieving changes in breakfast consumption may, however, require other strategies.

## Background

Unhealthy dietary habits, in terms of consuming insufficient fruits, consuming an unhealthy breakfast or skipping breakfast, and consuming too many high-fat snacks, are common among youngsters in the Western world [1,2] including the Netherlands [3-5]. Improvement of dietary intakes can prevent overweight and obesity and the prevalence of chronic diseases later in life [6]. Schools are considered an obvious setting for such health promotion initiatives since the target population can easily be reached there. Reviews have shown moderate evidence that school-delivered healthy diet programmes have effects on dietary intakes, such as fruit and vegetable intake and fat intake of children and adolescents [7-9].

Krachtvoer is a Dutch school-based healthy diet programme for 12- to 14-year-olds attending the first two years of prevocational education. The programme specifically targets prevocational schools, since these often incorporate a relatively large proportion of students from families with lower socio-economic positions (SEP) [10]. Students coming from families with lower SEP tend to have less healthy dietary habits than their peers from more privileged families [11,12]. The Krachtvoer programme aims at increasing the consumption of fruit, achieving a daily healthy breakfast, and decreasing the consumption of fats by replacing high-fat snacks (e.g. chocolate, potato chips) by non-fat or low-fat snacks [13]. The programme consists of eight lessons with a fixed content, but also some optional activities (see Additional file 1: Figure S1).

In contrast to existing nutrition education curricula, which aim to increase knowledge, the Krachtvoer programme was the first Dutch programme for this target group to aim at achieving behaviour change based on principles from behaviour change theories. It uses a combination of experiential learning activities (e.g. tasting healthy products) and cognition driven activities (e.g. comparing personal dietary intakes with the national recommendations). The programme builds on the three main phases in the Self Regulation Theory [14] successively aimed at raising awareness on personal dietary intakes, proposing solutions for not meeting Dutch dietary recommendations, and setting personal goals for dietary improvement [15,16]. The programme also incorporates insights from the literature on awareness [17], the Theory of Planned Behaviour [18], the Attitude-Social influence-Self Efficacy Model [19] and the action planning literature [20]. Examples of the methods and strategies derived from these theories are guided practice (preparing a fruitshake) and creating action plans (by means of a computer program) [15].

The Krachtvoer programme was first developed and evaluated in 2001 and revised in 2007 [15]. A clustered randomized controlled trial of the first version of the programme showed mixed effects on students’ dietary intakes within a month after programme implementation [3]. This study did not include a follow-up. Some effects were revealed in the whole group of students, others only in subgroups. The programme was effective in increasing fruit consumption in the group as a whole (mean difference experimental and control groups 0.12 servings of fruit a day).

As regards breakfast, no effects were found on breakfast frequency, but some beneficial effects were found in subgroups of students in terms of nutrients consumed at breakfast (i.e. saturated fat consumption at breakfast decreased among students with the highest baseline intakes, while vitamin C consumption increased for students with intermediate baseline consumption).

Mixed results for high-fat snack consumption were found in subgroups of students (i.e. the snacking frequency, the number of times that snacks had been consumed the day before and the total fat consumption from snacks decreased among students with highest baseline intakes, while the number of snacks consumed the day before increased among students with the highest baseline intakes).

Although the process evaluation of the first version of the programme yielded positive results in terms of programme appreciation and implementation, programme improvements were still recommended [16]. Nationwide dissemination required expanding the target group to include students from a lower educational subtrack within prevocational education and students of non-Dutch ethnicity [21,22]. For the students with more favourable baseline dietary intakes, who were already part of the target group, additional methods were needed to prevent opposite programme effects [3,16].

The development of health promotion programmes often ends after the first programme evaluation, although longer-term effects may not yet have been evaluated, and process findings may not have been incorporated in a revised version of the programme. We were unable to find any repeated evaluation studies with the aim to sequentially improve programme outcomes after initial field testing.

This current paper describes the evaluation of the revised version of the Krachtvoer programme. The process revision process and programme changes have been described elsewhere [15,21,22]. The first aim of this study was to determine if the revised Krachtvoer programme had resulted in increased fruit and fruit juice consumption, as well as daily healthy breakfast, and decreased consumption of high-fat snacks in the short- and longer-term. The second aim was to explore whether the effects varied in subgroups of students by gender, educational track, year of the class (first or second), and baseline dietary intakes.

## Methods

### Study design, sample, and data collection

Between February and June 2008 health promotion professionals from five Dutch Regional Public Health Services (RPHSs) spread over the Netherlands were asked to recruit a total of 25 schools out of 110 schools that did not participate in another study of the RPHS to limit research burden at schools. Although 10 schools in each condition would have been sufficient, 5 additional schools were included in order to react the level of power needed for an accompanying implementation study [22]. Schools had to offer education to at least 50 first- or second-year students in one of the three highest (of four) subtracks of prevocational education, for whom the programme was specifically developed. Targeting the lowest subtrack would require more practical programme strategies.

Recruitment took place in accordance with the steps of the adoption strategy [13]. Adopting schools were randomly assigned to the experimental or the waiting-list control condition by an independent researcher using a computerized random number generator. Teachers from experimental schools were asked to implement the Krachtvoer lessons over a period of eight weeks between September to December 2008, while control schools carried out the usual nutrition education curriculum aimed at increasing knowledge in the same period and postponed Krachtvoer implementation for one year. Teachers implemented the lessons in the first- and/or second-year classes in which the topic nutrition was scheduled for the school year 2008–2009. Students completed questionnaires individually as a classroom activity at baseline (1–4 weeks before implementation), at posttest (1–4 weeks after implementation), and at the repeated posttest (6 months after implementation). The teachers supervised the completion of the questionnaires. In case of objection students could fulfil another task during the lesson. The study was exempt from ethical review according to Dutch standards.

### Measures

Background variables included gender, country of birth of both parents, and postal code. Students’ ethnicity was defined as non-native if at least one of the parents had been born abroad [23]. Postal code was used as an indicator of SEP, based on a factor score (range −4 to 4 [high to low]) calculated over three SEP indicators (income, educational level and employment) of people living in different Dutch postal code areas [24,25]. Teachers provided data on school-related variables including educational track (lower [subtrack 2 of prevocational education] or higher [subtracks 3 and 4 of prevocational education] and year of the class (first of second).

One item regarding the number of days a week on which fruit was usually consumed, and one item regarding the number of servings of fruit consumed on these days were taken from a validated fruit and vegetable food frequency questionnaire (FFQ) [26]. Frequency and quantity scores were multiplied and divided by 7 to obtain the average fruit consumption per day. Two similar items were used to assess fruit juice consumption (glasses per day). Fruit juice was defined as juice of fruit without additional sugar. Finally, yesterday’s fruit consumption was measured by one item (i.e. the number of servings of fruit consumed the day before).

Breakfast consumption was measured with one FFQ item on the number of days a week on which breakfast was usually consumed. An open-ended recall item asking to describe all the foods and drinks consumed for breakfast that morning was used to assess breakfast consumption (yes/no) and as an indicator of breakfast quality in terms of nutrient intake.

A snack consumption FFQ item was derived from a validated fat consumption questionnaire [27], and asked for the number of days a week on which students usually consumed snacks (except fruit and vegetables) between meals, and the number of times snacks were consumed on these days. Examples of snacks were provided (e.g. chocolate, rice crackers). These two items were multiplied and divided by 7 to obtain the average number of times a day that snacks were consumed. Yesterday’s snack consumption was measured with one item (i.e. the number of times that snacks had been consumed the day before). Examples of snack portions were provided in the question (e.g. one small bag of potato chips, one candy bar). Finally, five items were used to measure the usual consumption of snacks from five snack groups: sweets (e.g. chocolate, acid drops), savoury snacks (e.g. potato chips, popcorn), ice-creams (e.g. soft ice-cream, water ice), fried snacks (e.g. minced-meat hot dog, Vietnamese spring roll), and cookies (e.g. chocolate cookies, fibre cookies). In line with the national recommendations [28], favourable products and unfavourable products were distinguished within each snack group (e.g. water ice versus soft ice-cream). Frequency items were formulated for each snack group (e.g. which category of ice-creams do you usually consume?: “I do not eat ice-cream”, “I usually eat [followed by examples of favourable products, such as water ice],” I usually eat “[followed by examples of unfavourable products such as soft ice-cream],” and “I consume ice-creams from both categories equally”).

### Statistical analyses

Since fruit and fruit juice frequency variables were not normally distributed, the data were log-transformed. The breakfast frequency variable was extremely skewed and therefore dichotomized based on the dietary recommendations (daily consumption [coded as 1] versus less than seven days [coded as 0]). Two types of analysis were performed on the open-ended recall item of that mornings’ breakfast consumption. First, we coded whether students had eaten breakfast (yes/no). Second, total energy intake and nutrients (total fat, saturated fat, carbohydrates, proteins, and fibres) were calculated using the nutrients calculation Eetmeter programme (Voedingscentrum, Den Haag, Netherlands), and log-transformed.

The snack frequency variables were log-transformed. Further, the responses to the five questions on the usual consumption of sweets, cookies, savoury snacks, ice-cream and fried snacks were each dichotomized into a value for “favourable”(coded as 1 and including those who reported no consumption of this type of snack and those reporting their product choices to be more often favourable than unfavourable) and a value for “unfavourable” (coded as 0 and including those who reported more frequent unfavourable than favourable product choices and those who reported equally frequent consumption of favourable and unfavourable products). Mixed linear regression analyses were conducted, in which the programme accounted for missing data based on the observed data [29]. The model disregards missing observations at that time point and corrects for nested designs by including random intercepts. Mixed linear and mixed logistic regression were conducted using PASW Statistics 17 (SPSS Inc. Chicago, IL) for continuous outcomes and MLwiN version 2.02 (Centre for Multilevel Modelling, Bristol, UK) for binary outcomes.

Baseline differences between the intervention and control conditions were assessed with mixed logistic regressions with condition as the outcome variable (control = 0, experimental = 1), and with student background variables (gender, educational track, year, ethnicity and SEP) and baseline dietary intake variables (fruit, fruit juice, snack, breakfast) as fixed factors, and with a random intercept of class. Selective dropout was tested using comparable analyses with dropout at the posttests as outcome variables. An 0.05 significance level was used for these analyses.

The short- and longer-term intervention effects were analysed using mixed linear and mixed logistic regression including a random intercept at the student and class levels. The dependent variables were the primary outcomes on fruit, fruit juice, breakfast, and snack intakes. Short- and longer-term intervention effects on these outcomes were examined by including interactions between posttest and condition, and by including the separate fixed factors of gender, educational track, year, baseline dietary intake, ethnicity and SEP. P-values below 0.05 were considered significant. Subgroup analyses were done for both posttest values if there were significant moderators of posttest values, condition and one of the predictors of gender, educational track, year, and dietary intakes at baseline (baseline intakes below mean consumption were coded as 0, baseline intakes above mean consumption were coded as 1). The final model of each individual subgroup analysis included one of the outcomes in terms of fruit, fruit juice, breakfast and snack intakes as dependent variables. Independent variables were the interactions between posttest values and condition, and the individual fixed factors of gender, educational track, year, baseline dietary intake, ethnicity and SEP (excluding the variable for which the specific subgroup analysis was performed).

Since our randomization was not completely successful, all analyses of main and subgroup programme effects were repeated using only the schools randomized according to protocol, to prevent distortion of programme effects.

## Results

### Response rates, baseline differences and missing values

A total of 25 schools with 2097 students participated in the study. Twenty-two of the 25 schools were randomly assigned to the control condition (N = 8) or the experimental condition (N = 14). Three others were assigned to the control condition at their own request, since one school had already ordered other teaching materials, a second school was already participating in an alcohol prevention programme, and the last school had to invest in activities to stop the decline in student numbers. One experimental school with 222 students was excluded, since it had not implemented the Krachtvoer programme due to logistical problems at the school. The number of experimental schools was higher than that in the control condition, to increase the power of an accompanying implementation study [22].

The final sample for analysis consisted of 13 experimental schools with 53 classes and 1117 students, and 11 control schools with 38 classes and 758 students. Compared to the control condition, the experimental condition included more second-year students (OR = 2.62; p < 0.05) and more students attending the higher educational subtracks (OR = 3.60; p < 0.001). Dropout numbers at the first posttest were 89 for the experimental condition and 77 for the control condition, and those at the second posttest 101 and 76, respectively. Reasons for drop-out were absence from the lesson in which the questionnaire was completed and incomplete background characteristics which made it impossible to link the separate measurements. Drop-out was not selective.

The final population had a mean age of 12.9 years. Most students attended the higher educational subtracks (65.9%) and were in second year (58.9%). Students were representative of the Dutch prevocational student population with just over half of participants being female, 80% being of Dutch ethnicity, and a mean SEP comparable to the average Dutch score (Table 1).

**Table 1:** Baseline scores for background characteristics and differences between control (n = 758) and experimental condition (n = 1117)

	**Total group**	**Control condition**	**Experimental condition**	**Odds ratio (CI) baseline difference**^**1**^
	**uncorrected %**	**uncorrected %**	**uncorrected %**	**baseline difference^1^**
Gender				1.024
- Boys	52.3	42.2	51.4	(0.831-1.263)
- Girls	47.7	57.8	48.6	
Ethnicity				0.987
- Dutch	80.4	81.8	79.4	(0.756-1.289)
- Other	19.6	18.2	20.6	
Year				2.617*
- First year	41.1	50.9	34.4	(1.050-6.523)
- Second year	58.9	49.1	65.6	
Educational track				3.597**
- Theoretical subtracks of prevocational education and senior general education (higher subtracks)	65.9	50.5	76.3	(1.412-9.161)
- Practical subtrack of prevocational education (lower subtrack)	34.1	49.5	23.7	
	**Total group uncorrected mean (SD)**	**Control condition uncorrected mean (SD)**	**Experimental condition uncorrected mean (SD)**	***t*-test (CI) baseline difference**
Mean factor score for socio-economic position (SD) (range -4 to 4)	-0.04 (0.86)	-0.01 (0.91)	-0.04 (0.86)	1 (0.874-1.145)

Most missing values were related to the snack category items (20% missing values for sweets, savoury snacks, biscuits, ice-creams, and 21% missing values for fried snacks). None of the other outcome items had more than 15% missing values.

### Effects on fruit, breakfast, and snack consumption

Table 2 shows the short- and longer-term effect estimates for the continuous outcome measures of fruit, fruit juice, nutrients consumed at breakfast, and snack consumption. Table 3 shows the short- and longer-term effect estimates for the dichotomous outcome measures of breakfast frequency and snack consumption. Table 4 presents significant intervention effects in subgroups.

**Table 2:** Results of the mixed linear analyses of continuous outcomes of fruit, snacks, and breakfast consumption

	**Experimental condition**			**Control condition**			**B short-term effect**^**1**^	**B longer-term effect**^**1**^
	**uncorrected mean (SD)**			**uncorrected mean (SD)**				
	**T0**	**T1**	**T2**	**T0**	**T1**	**T2**		
	**(n = 1117)**	**(n = 1028)**	**(n = 1016)**	**(n = 758)**	**(n = 681)**	**(n = 682)**		
**Fruit**								
Fruit frequency	0.98	1.13	1.03	1.11	1.11	1.01	0.048***	0.033***
(servings a day)	(0.80)	(0.81)	(0.81)	(0.86)	(0.92)	(0.85)	(0.023-0.053)	(0.017-0.048)
Yesterday’s fruit consumption	0.97	1.23	1.18	1.02	1.16	1.08	0.023*	0.026*
(servings)	(0.90)	(0.94)	(0.98)	(0.86)	(0.99)	(0.92)	(0.002-0.044)	(0.005-0.047)
Fruit juice frequency	1.00	1.09	1.06	1.02	0.98	1.01	0.022*	0.013
(glasses a day)	(0.95)	(0.96)	(0.98)	(0.97)	(0.94)	(0.96)	(0.004-0.041)	(-0.005-0.032)
**Snacks**								
Snack consumption frequency	1.90	1.86	1.84	1.86	1.90	1.87	0.001	-0.007
(times a day)	(1.57)	(1.50)	(1.56)	(1.51)	(1.67)	(1.69)	(-0.020-0.020)	(-0.026-0.013)
Yesterday’s snack consumption	1.97	2.09	1.99	1.99	2.11	2.04	0.014	0.004
(number of snacks)	(1.51)	(1.59)	(1.60)	(1.54)	(1.74)	(1.74)	(-0.011-0.039)	(-0.021-0.030)
	**T0**	**T1**	**T2**	**T0**	**T1**	**T2**	**B short-term effect**^**1**^	**B longer-term effect**^**1**^
	**(n = 769)**	**(n = 769)**	**(n = 724)**	**(n = 486)**	**(n = 484)**	**(n = 467)**		
**Breakfast**^**2**^								
Energy	302.9	300.7	302.4	314.3	299.4	298.0	0.023	0.027
(kcal)	(163.9)	(146.2)	(144.4)	(169.0)	(158.3)	(154.7)	(-0.005-0.051)	(-0.001-0.056)
Fat	9.14	9.30	9.42	9.48	9.29	9.48	0.013	0.013
(grams)	(7.44)	(7.08)	(6.96)	(8.01)	(7.33)	(7.70)	(-0.030-0.055)	(-0.032-0.058)
Saturated fat	4.35	4.47	4.55	4.61	4.60	4.80	0.005	0.006
(grams)	(3.87)	(3.90)	(3.80)	(4.35)	(4.19)	(4.29)	(-0.039-0.046)	(-0.037-0.049)
Carbohydrates	40.93	40.14	40.26	42.10	40.02	39.06	0.025	0.039
(grams)	(24.41)	(19.90)	(19.64)	(21.82)	(21.00)	(20.75)	(-0.005-0.054)	(0.008-0.069)*
Protein	11.72	11.41	11.50	12.06	11.51	11.68	0.006	0.007
(grams)	(7.32)	(6.75)	(6.81)	(8.14)	(7.43)	(7.17)	(-0.025-0.037)	(-0.025-0.039)
Fiber	2.87	2.85	2.81	3.03	2.77	2.84	0.035	0.041
(grams)	(2.53)	(2.45)	(2.05)	(2.11)	(2.03)	(2.26)	(0.005-0.064)*	(0.011-0.072)**

^1^Reported intervention effects were corrected for a random intercept of measurement, student, and class, and the fixed factors of gender, year, educational track, SEP, and ethnicity * P < 0.05; **P < 0.01; ***P < 0.001.

^2^ Breakfast nutrients were calculated for students who had consumed breakfast.

**Table 3:** Results of the mixed logistic regressions of dichotomous outcomes of snacks and breakfast consumption

	**Experimental condition**			**Control condition**			**Odds Ratio for short-term effect**	**Odds Ratio for longer-term effect**
	**uncorrected %**			**uncorrected %**				
							**(CI)**^**1**^	**(CI)**^**1**^
	**T0**	**T1**	**T2**	**T0**	**T1**	**T2**		
	**(n = 1117)**	**(n = 1028)**	**(n = 1016)**	**(n = 758)**	**(n = 681)**	**(n = 682)**		
**Breakfast consumption**								
Breakfast frequency							1.10	1.17
							(0.79-1.54)	(0.83-1.64)
-Daily	79.2	73.7	76.3	77.2	74.1	75.3		
-Less than seven days	20.8	26.3	23.7	22.8	25.9	24.7		
Consumed breakfast that morning							0.81	0.79
							(0.53-1.26)	(0.52-1.20)
-Yes	88.0	87.6	84.7	90.2	85.8	83.5		
-No	12.0	12.4	15.3	9.8	14.2	16.5		
**Snack consumption**								
Sweets consumption							1.36*	1.48**
							(1.02-1.81)	(1.11-1.97)
-Favourable category	39.1	42.1	44.6	47.6	41.4	43.3		
-Unfavourable category	60.9	57.9	55.4	52.4	58.6	56.7		
Savoury snacks consumption							1.66**	1.16
							(1.22-2.26)	(0.85-1.57)
-Favourable category	28.2	30.9	29.0	34.8	26.9	31.8		
-Unfavourable category	71.8	69.1	71.0	65.2	73.1	68.2		
Ice-cream consumption							1.55**	1.29
							(1.15-2.09)	(0.97-1.74)
-Favourable category	32.6	38.5	40.3	37.9	32.2	37.7		
-Unfavourable category	67.4	61.5	59.7	62.1	67.8	62.3		
Fried snack consumption							1.58**	1.29
							(1.12-2.22)	(0.92-1.81)
-Favourable category	64.9	61.5	61.8	64.0	62.1	59.1		
-Unfavourable category	35.1	38.5	38.2	36.0	37.9	40.9		
Cookies consumption							0.90	1.01
							(0.67-1.20)	(0.75-1.36)
-Favourable category	64.9	61.5	61.8	64.0	62.1	59.1		
-Unfavourable category	35.1	38.5	38.2	36.0	37.9	40.9		

^1^Reported intervention effects were corrected for a random intercept of measurement, student, and class, and the fixed factors of gender, year, educational track, SEP, and ethnicity * P < 0.05; **P < 0.01; ***P < 0.001.

**Table 4:** Intervention effects in subgroups on continuous outcomes regarding fruit, snack, and breakfast intakes, comparing the experimental group (n = 1117) with the control group (n = 758)

	**Intervention effects among first and second year students**				**Intervention effects among students with more and less favourable baseline dietary intakes**				**Intervention effects among lower and higher educational subtracks**	
	**First-year**	**Second-year**	**First-year**	**Second-year**	**Low baseline intakes**	**High baseline intakes**	**Low baseline intakes**	**High baseline intakes**	**Lower educational subtrack**	**Higher educational subtracks**
	**Short-term effects B (CI)**^**1**^		**Longer-term effects B (CI)**^**1**^		**Short-term effects B (CI)**^**1**^		**Longer-term effects B (CI)**^**1**^		**Short-term effects B (CI)**^**1**^	
**Fruit**										
Fruit juice frequency	-	-	-	-	-	0.034**	-	-	-	-
(glasses a day)						(0.009-0.059)				
**Breakfast**^**2**^										
Total energy	0.056*	-	0.053*	-	-	-	-	-	-	0.044*
(kcal)^1^	(0.013-0.098)		(0.012-0.094)							(0.008-0.080)
Total fat	-	-	0.072*	-	-	-	-	-	-	-
(grams)			(0.008-0.137)							
Total saturated fat	-	-	0.064*	-	-	-	-	-	-	-
(grams)			(0.003-0.126)							
Total carbohydrates	-	-	-	-	-	-	-	-	-	0.046*
(grams)										(0.007-0.086)
Total fiber	0.085***	-	-	-	-	-	0.043*	-	-	-
(grams)	(0.041-0.129)						(0.006-0.081)			
	**Short-term OR**		**Longer-term OR**		**Short-term OR**		**Longer-term OR**		**Short-term OR**	
	**First-year**	**Second-year**	**First-year**	**Second-year**	**Unfavourable baseline intakes**	**Favourable baseline intakes**	**Unfavourable baseline intakes**	**Favourable baseline intakes**	**Lower educational subtrack**	**Higher educational subtracks**
**Snacks**										
Ice-cream	-	-	-	-	10.00***	0.12***	-	-	0.59*	**-**
					(6.92-14.5)	(0.08-0.18)			(0.38-0.91)	

^1^Reported intervention effects were corrected for a random intercept of measurement, student, and class, and the fixed factors of gender, year, educational track, SEP, and ethnicity.

^2^Breakfast nutrients were calculated for students who had consumed breakfast.

* P < 0.05; **P < 0.01; ***P < 0.001.

Short- and longer-term favourable intervention effects were found for fruit frequency and yesterday’s fruit consumption. A significant short-term increase in fruit juice consumption was found in the experimental group (Table 2), attributable to an effect among students with a higher baseline frequency (Table 4).

No intervention effects were found for the breakfast frequency item or the percentage of students who had consumed breakfast that morning (Table 3). A favourable short- and longer-term intervention effect on fibres was seen for students who had eaten breakfast (Table 2). The short-term effect was attributable to a favourable effect among the subgroup of first-year students, while the longer-term effect was attributable to a favourable effect among students with the lowest fibre intakes at baseline (Table 4). A short-term effect on carbohydrates was seen among students attending the higher educational subtracks (Table 4), and a longer-term effect among the whole group (Table 2). Energy intake at breakfast increased significantly among first-year students, in both the short and longer-term, and in the short term among students attending the higher educational subtracks. A significant increase in fat and saturated fat intake was seen among first-year students (Table 4).

The outcome variables regarding snack frequency and yesterday’s snack consumption did not show any effects (Table 2). Some favourable effects were revealed regarding the categories of snacks consumed (Table 3), including short- and longer-term effects on sweets consumption and short-term effects on the consumption of savoury snacks, ice–creams, and fried snacks. Students who had eaten items from the unhealthy ice-cream category at baseline showed a beneficial effect in the short term, while the opposite was found for students who had consumed products from the healthier ice-cream category at baseline (Table 4). The intervention had an adverse short-term effect on ice-cream consumption among students from the lower educational subtrack (Table 4). The consumption of cookies did not change significantly as a result of the intervention (Table 3).

### Repeated analyses without the non-randomized control schools

In general, the repeated analyses without the non-randomized control schools revealed fewer significant programme effects.

As regards the continuous outcomes (Table 2), the short- and longer-term effect on fruit consumption remained. As regards yesterday’s fruit consumption, the short-term effect remained, which could be explained by a new subgroup effect among first-year students. The main longer-term intervention effect on yesterday’s fruit consumption was no longer found, but new subgroup effects were revealed among girls (increase) and boys (decrease). As regards fruit juice consumption, the short-term main intervention effect and the subgroup effect among students with higher baseline intakes remained, but the longer-term main intervention effect was no longer significant. As regards snacks, the continuous outcomes of snack consumption still showed no intervention effects. As regards breakfast consumption, the main short- en longer-term intervention and subgroup effects were no longer significant.

As regards the dichotomous outcomes (Table 3), we still found no main intervention or subgroup effects on breakfast consumption. As regards snack consumption, the main intervention effects on categories of snacks consumed remained, apart from the short-term effect on sweets consumption. The subgroup effects on ice-cream consumption were no longer found.

## Discussion

This paper reports on the effect evaluation of the revised Dutch healthy diet programme called Krachtvoer. Favourable effects of the intervention were found in the experimental group as a whole, though some mixed intervention effects emerged in subgroups.

The favourable intervention effects on fruit consumption were comparable to those found in the effect evaluation of the first version of the programme [3], but the present study also enabled us to show some longer-term effects. Additionally, the present study found short-term effects on fruit juice consumption. Only two other secondary school programmes reported intervention effects on fruit consumption [30,31].

Just as in the first version, we found no effects on breakfast frequency which could be attributed to relatively high baseline rates. We found limited effects on fibre and carbohydrate intakes, which could be attributable to higher rates at baseline in the control condition. We found some additional intervention effects in subgroups regarding nutrients consumed at breakfast, but we also found some worrying effects, especially among first-year students. The limited effects on breakfast consumption may be attributable to numerous causes. First, there were large variations in the specificity of students’ answers on the open-ended item about that morning’s breakfast, which may have hampered the detection of changes at nutrient level. Second, students already had relative favourable baseline values for breakfast intakes. In fact, the percentage of Dutch youngsters who have breakfast each day is higher than that in other European countries [1]. Our results show that the mean energy intake at breakfast is around 300 kcal, which is in line with recommendations [32]. Since skipping breakfast is related to a less healthy general food pattern [33,34] (e.g. consuming snacks late in the evening resulting in not being hungry in the morning) it may be more resistant to change than other nutrition behaviours. We found only one other healthy diet intervention study at secondary schools showing an increase (0.5 to 0.7) in the number of days a week on which cereals were eaten at breakfast [31]. However, baseline consumption in that specific target group was much lower and therefore the results of the two studies are difficult to compare. Others found no programme effects on breakfast intakes of secondary school students [35,36]. A study among Dutch students aged 10 to 18 showed that breakfast frequency decreases strongly from the age of 14 to 15 years onwards [4]. Therefore, our intervention might be more effective among an older age group. Overall, the lack of effects on breakfast consumption in our study implies that these lessons should be removed from a new version of the programme. We recommend re-examining the epidemiological data on the most problematic modifiable health-related dietary behaviours, and replacing the breakfast topic by a more problematic one.

In line with the effect evaluation of the first version [3] we found no main intervention effects on snack frequency; subgroup effects from the first effect evaluation were not found this time. In agreement with the purpose of the programme, significantly more students in the experimental condition reported consuming snacks from the favourable food categories (healthier choice or no snacks at all) at the first and/or second posttest. It is striking that the analyses for subgroups of students based on educational track and baseline dietary intakes revealed that students from the lower educational subtrack and students who consumed ice-creams from the more favourable categories at baseline had unfavourable short-term intervention effects, while students consuming from the unfavourable ice-cream category had beneficial short-term effects from the intervention. Others have reported mixed effects of health education in secondary schools, including beneficial changes in fat consumption during the day [37], reduced sugars and sweets during the day [38], but also a lack of effect on snack intakes [39]. A Dutch study revealed that in-class education about snacks and soft drinks, accompanied by changes to the snack and soft drink vending machines, new product labels on snacks and soft drinks, and decreased price of low-calorie products led to higher sales of lower-calorie products in secondary schools [40]. Together with the findings of the current study, this indicates that healthier snack choices can be achieved by healthy diet interventions.

Limitations of our study include the fact that we had to randomize students at school level. Our experimental condition included relatively more second-year students, while our control condition included more lower-educated students. Both characteristic are negatively related to eating habits. Although we tried to correct for this by including all background characteristics in all of our analyses, it may have still have influenced the results. Further, our randomization was not completely successful and this may have influenced our results. We performed the analyses with and without three non-randomized control schools. Although some programme effects were no longer found, most main intervention effects were comparable to those in the analyses that included all schools. Most changes occurred in subgroups, most likely because of power issues. The impact of Krachtvoer at population level when it comes to broader dissemination is also determined by schools that do adopt, but not implement the programme. It is a flaw of the current study that one non-implementing school in the intervention condition dropped out. This study only reveals the programme effects in case the programme is actually implemented. Effects will undoubtedly be diluted when it comes to real-life dissemination. The study did however include schools with different levels of implementation, which will also be the case during real-life dissemination.

Another limitation is that we used a combination of existing questions from validated questionnaires and added new categorical snack items. Most of the items were used in the previous effect evaluation study among the same target group [3], but no validation study of the questionnaire as a whole has been performed among our group of prevocational students and no tested questionnaires were available.

With regard to fruit consumption, we measured fruit and fruit juice consumption separately by means of frequency items per week, so we cannot generalize our results to the general fruit recommendation per day (replacing at most one serving of fruit by fruit juice each day). Since mean fruit juice consumption was slightly above the national dietary recommendation, the goal of increasing fruit consumption is considered relevant for half of the group of students, who consumed less than one glass of fruit juice a day.

With regard to snack consumption we were faced with high non-response rates of up to 21% on our snack category items. Possibly these items were too difficult, so these items need further development and testing.

Other limitations are the fact that our follow-up test took place only six months after programme implementation, and the fact that the use of self-reports by self-administered questionnaires may be problematic especially in studies aiming to identify effects on nutrients consumed at breakfast, as discussed above. A final limitation is that the number of students of non-Dutch ethnicity in our study population was too small to allow specific intervention effects in this subgroup to be examined, although we did adjust the programme by including information on culture-specific events (e.g. Ramadan), habits, and food products.

Programme-related limitations include implementation problems. Our process evaluation showed that programme implementation was good, except for the implementation of the final two lessons aimed at translating positive behavioural intentions into actions which were implemented by the fewest teachers (61%) [15]. Additional dose response analyses may reveal whether this indeed caused the limited programme effects.

Strengths of our study include the study design, the high response rates, the statistical analyses performed, and the thorough assessment of effect moderators. The current study revealed some promising effects as a result of the improved programme in a wider population of lower-educated students, but further improvements are still possible. Programme revisions were successful and the repeated effect evaluation provided new information on the longer-term intervention effects and the effects among particular subgroups of students, while some effects in the total group changed as well. The dietary changes that we found are small, but even such small changes can contribute to changes in disease risk [5,41] if implemented on a large scale.

## Conclusion

It is important to continually update promising health promotion programmes such as Krachtvoer and to conduct repeated evaluation studies. Subgroup analyses can help us to detect unfavourable and favourable subgroup effects of interventions and provide recommendations for programme revisions. We conclude that Krachtvoer was not successful in changing breakfast habits, which was relatively good in our target group. The breakfast lessons should therefore be excluded from the next version of the programme and substituted by a more problematic dietary behaviour. We were able to achieve some favourable main intervention effects in terms of fruit and snack consumption in a relevant target group attending lower prevocational schools. Based on the results of the current effectiveness study, we propose that the fruit and snack lessons be disseminated nationwide. We currently have no information on the cost-effectiveness of the Krachtvoer programme, so we recommend a further cost-effectiveness analysis. The effects on dietary behaviours might be optimized by implementing Krachtvoer as a component of a more comprehensive whole-school approach, targeting environmental influences as well [42-44].

## Competing interests

The authors declare that there are no conflicts of interest.

## Authors’ contributions

KB, PVA, MM, TP and NDV contributed to the design of the study. KB and LR contributed to the conduct of the study, and monitored recruitment and data collection. KB and MDR performed statistical analyses. KB and PVA prepared a draft of the manuscript. All authors contributed to reviewing and improving the manuscript in terms of content and approved the final manuscript.

## Supplementary Material

Additional file 1 **Figure S1.** The Krachtvoer programme. Click here for file
