# Supplementary material for: Healthier food choices as a result of the revised healthy diet programme Krachtvoer for students of prevocational schools
Source: Int J Behav Nutr Phys Act. 2012 May 24;9:60. doi: 10.1186/1479-5868-9-60 (PMC3544148; doi:10.1186/1479-5868-9-60)
Supplement: Additional file 1 — Figure S1. The Krachtvoer programme. [file 1479-5868-9-60-S1.doc]

**The Krachtvoer programme**

*Lessons*

The programme covers 8 lessons and one optional pre-programme lesson on nutrients. Some slots in the lessons 4 to 8 can be filled by 7 optional activities (of which at least 2 should be used).

- Before the start of the programme:
  - **Lesson 0: Nutrients.** Students learn about nutrients (e.g. saturated fats, carbohydrates), their functions in the human body, and food products containing these nutrients
- Phase 1. Students are made aware of their own dietary intake and deviations from the Dutch dietary guidelines.
  - **Lesson 1: Nutrition, foods and health.** Students’ knowledge on fruit, breakfast and high-fat snacks is tested in a quiz. Students receive a lunchbox with a flyer and three healthy food items representing the three topics.
  - **Lesson 2: Fruit and fruit juices.** Students learn to read fruit juice labels. Knowledge about the differences between fruit juices and other juices is tested with a fruit juice test. They are made aware of their own fruit or fruit juice consumption, and become familiar with the national recommendations of consuming two servings of fruit a day (of which one can be replaced by a fruit juice).
  - **Lesson 3: Breakfast and snacking***.* Students are made aware of their own breakfast habits and high-fat snack consumption. The disc of five and an overview of low- and high-fat snacks are discussed and compared with their own breakfast and snack intakes.
- Phase 2. Students specify reasons for deviations and are encouraged to propose solutions.
  - **Lesson 4: Barriers to healthy eating.** Students answer questions on personal reasons for (not) meeting the dietary recommendations on fruit, breakfast and snack intakes, and give each other tips on healthy eating. Students receive a **take-home bag** with healthy products, a newsletter with tips and recipes, and a notepad designed to involve their parents.
  - **Optional activities in lesson 4 (part 2) and lesson 7 (part 1)**
    - **National recipe contest**. Students participate in a national recipe contest.
    - **Magazine**. Students work with a magazine offering information, tests, puzzles, a horoscope, role model stories and healthy recipes.
    - **Website**. Students visit the Krachtvoer website to read information, do a snack test (i.e. distinguishing between high- and low-fat snacks) and a knowledge test (e.g. practical and theoretical knowledge about fruit, breakfast and snacks), and send e-cards.
  - **Lesson 5. Food exposure.**
    - **Taste testing**. Students judge products by tasting, smelling and looking at (unfamiliar) fruit, breakfast products and favourable snacks.
    - **Fruit tasting**. Students bring fruits from home to school and taste them together.
    - **Preparing a fruit shake**. Students prepare a healthy tasty fruit shake
  - **Lesson 6. Advertisements**. Advertising tricks are discussed and are applied in an advertising poster produced by the students.
- Phase 3. Students implement and evaluate an action plan.
  - **Lesson 7 part 2: Personal action plans**. Students use a program on the website to generate a personal action plan (what, when, where) to improve their fruit, breakfast or snack intake during the next week.
  - **Lesson 8: Evaluation of personal plans**. In-class evaluation of the action plans, followed by an optional activity.

*Programme materials*

- Students’ workbook, posters, postcards, a lunchbox with healthy food items, a magazine, a website with a computer program for action plans, a take-home bag for parents, containing a newsletter and healthy food items, a recipe contest and a teacher manual.
